# A High Neutrophil-to-Lymphocyte Ratio Predicts Higher Risk of Poststroke Cognitive Impairment: Development and Validation of a Clinical Prediction Model

**DOI:** 10.3389/fneur.2021.755011

**Published:** 2022-01-17

**Authors:** Fei Zha, Jingjing Zhao, Cheng Chen, Xiaoqi Ji, Meng Li, Yinyin Wu, Li Yao

**Affiliations:** Intensive Care Unit, The Second People's Hospital, Hefei, China

**Keywords:** cognitive impairment, neutrophil to lymphocyte ratio (NLR), poststroke cognitive impairment, prognosis, nomogram

## Abstract

**Objective:**

Poststroke cognitive impairment (PSCI) is a serious complication of stroke. The neutrophil-to-lymphocyte ratio (NLR) is a marker of peripheral inflammation. The relationship between the NLR and PSCI is far from well studied, and the thesis of this study was to assess the predictive value of the NLR in patients with PSCI, and establish and verify the corresponding prediction model based on this relationship.

**Methods:**

A total of 367 stroke patients were included in this study. Neutrophils, lymphocytes, and NLRs were measured at baseline, and clinical and neuropsychological assessments were conducted 3 months after stroke. The National Institutes of Health Scale (NIHSS) was used to assess the severity of stroke. A Chinese version of the Mini Mental State Examination (MMSE) was used for the assessment of cognitive function.

**Results:**

After three months of follow-up, 87 (23.7%) patients were diagnosed with PSCI. The NLR was significantly higher in PSCI patients than in non-PSCI patients (*P* < 0.001). Patient age, sex, body mass index, NIHSS scores, and high-density lipoprotein levels also differed in the univariate analysis. In the logistic regression analysis, the NLR was an independent risk factor associated with the patients with PSCI after adjustment for potential confounders (OR = 1.67, 95%CI: 1.21–2.29, *P* = 0.002). The nomogram based on patient sex, age, NIHSS score, and NLR had good predictive power with an AUC of 0.807. In the validation group, the AUC was 0.816.

**Conclusion:**

An increased NLR at admission is associated with PSCI, and the model built with NLR as one of the predictors can increase prognostic information for the early detection of PSCI.

## Introduction

Stroke is the second most important cause of declining acquired cognitive ability and dementia worldwide, with a high rate of disability (1–3). The incidence of poststroke cognitive impairment (PSCI) varies from 17–92% per year (4–6). As people pay more attention to disease and health, cognitive and psychological hazards are receiving increasing attention. Therefore, the recognition and early diagnosis of PSCI are of great significance for the functional recovery of stroke patients.

The existing body of research on PSCI has been reported, including white matter changes, brain microbleeds, silent infarcts, and lacunes (7–9). In addition, age, years of education, and National Institutes of Health Scale (NIHSS) scores were also reported (10–13). Neurodegenerative and vascular mechanisms can cause significant increases in inflammatory markers, including C-reactive protein, IL-1, IL-18, IL-6, and TNF-α, which may be the reason for PSCI (14, 15). Extensive studies have also shown that inflammatory pathophysiology plays a crucial role in regulating cognitive impairment (16–18). However, as an inflammatory marker, studies on the effects of the neutrophil-to-lymphocyte ratio (NLR) on PSCI are limited (19).

The NLR is a peripheral inflammatory marker that is freely received from circulation (10). Recently, the NLR has become a research hotspot for various diseases, such as cardiovascular diseases (20, 21), cancer (22, 23), and some inflammatory diseases (24). Existing studies have also have recognized the critical role played by the NLR in cognitive impairment and dementia. Studies have illustrated that the NLR has high predictive value in patients with Alzheimer's disease, and is also associated with cognitive dysfunction after carotid endarterectomy (25, 26). To date, the attention paid to the NLR and PSCI has been far from sufficient. The NLR, as an inflammatory marker, is associated with cognitive impairment (25, 26). There are few studies on the association between the NLR and PSCI, except for the study by Minwoo Lee et al. (19) who found that the NLR in acute ischemic stroke was independently associated with PSCI at 3 months poststroke. Nomograms have been widely used to predict the prognosis of oncology patients, predicting patient survival based on a simple score. So far, no nomogram has been developed for PSCI. The aim of this study is to further deepen the research on the relationship between the NLR and PSCI and to build a relevant nomogram to predict the probability of stroke patients developing PSCI. This prediction model was not previously available.

## Materials and Methods

### Subjects

This study was approved by the Ethics Committee of the Second People's Hospital of Hefei, and conformed to the Helsinki Declaration. From January 2012 to January 2017, 367 stroke patients were admitted to the hospital within 7 days of onset. The criteria for selecting the subjects were as follows: (1) patients aged between 18 and 80 years; (2) patients with diagnoses confirmed by computerized tomography (CT) or magnetic resonance imaging (MRI) at the time of admission; (3) patients with complete information including neutrophil and lymphocyte levels; and (4) patients who completed the Chinese version of the Mini Mental State Examination (MMSE) assessment at the 3 month follow-up at hospital discharge.

The criteria for excluding the subjects were as follows: (1) patients with acute or chronic inflammatory disease; (2) patients with transient ischemic attack; (3) patients who cannot be assessed for severe aphasia or dysarthria, or visual or auditory impairments at baseline or at follow-up (demonstrated by the exclusion of certain patients with severe stroke, high NIHSS scores); (4) patients with Parkinson's disease, severe cognitive impairment, or dementia; and (5) factors that severely affect inflammation indicators, including severe infection or antibiotic use before admission, blood disease, immunosuppression use, glucocorticoid use, or severe liver and/or kidney disease, as well as recent trauma and/or major surgery.

### Clinical Measurements

Patient baseline data include sex, age, body mass index (BMI), years of education, drinking history and smoking history, hypertension history, diabetes history, hyperlipidemia history, coronary artery disease history, and etiology of stroke. The patients underwent CT or MRI examination within 72 h of admission. Inflammation is associated with many diseases, and eliminating the inflammatory response of these diseases is very important for the study. According to the classification of ICD-10, diseases are divided into 21 categories. We considered endocrine, nutritional, and metabolic diseases, diseases of the circulatory system, mental and behavioral disorders, and diseases of the nervous system in the study in order to clarify the impact of inflammation associated with patients' complications on PSCI (these are the most frequently used diseases in previous studies, and the incidence rate is relatively high).

### Assessment

After admission and 3 months after discharge, trained neurologists used the MMSE to assess cognitive function. Studies have proven that the MMSE has high sensitivity and specificity for the diagnosis of cognitive impairment in the community setting and primary hospitals (23). Lower MMSE scores indicate more serious cognitive impairment. Due to the specific application of the cultural and social background of the Chinese elderly population, PSCI was defined as MMSE score ≤19 (illiterate), ≤22 (primary education), and ≤26 (secondary and above) depending on the patient's years of education (27, 28). The NIHSS is usually completed by two physicians independently within 8 h of admission, followed by a score comparison, with a third physician intervening to assess if there is a discrepancy. We used the NIHSS to assess the severity of stroke.

### Laboratory Test

In our hospital's laboratory, the researchers were blinded to the clinical outcomes and measured neutrophil and lymphocyte counts. The NLR was defined as the absolute neutrophil count divided by the absolute lymphocyte count within 24 h of hospital admission.

### Statistical Analyses

Continuous variables are expressed as the mean ± standard deviation and categorical variables are presented in terms of frequencies and percentages. Univariate analysis was performed by Student's *t* test (if the data did not obey a normal distribution, a Kruskal-Wallis test was used) or the chi-square test. Factors with a *P* < 0.05 were then analyzed by multivariate logistic regression. In multivariate logistic regression, we divided all stroke participants into PSCI and non-PSCI groups at the 3 month follow-up, adjusted for potential confounding factors, and analyzed the odds ratios (ORs) and 95% confidence intervals (CIs) for PSCI risk. To better reflect the clinical significance of changes in variables, we standardized the continuous variables in the logistic regression. The OR obtained in this way means that the probability of PSCI is several times higher/lower than before for the increase of one standard deviation unit. The variables for which we obtained a *P* < 0.05 by multivariate logistic regression were used as one of the predictors in the prediction model.

The correlation between the NLR and stroke severity (NIHSS score) was tested using the Pearson correlation test, and the correlation between the NLR and the possibility of PSCI occurrence was demonstrated using a continuous probability curve.

The nomogram used the ROC curve to assess the accuracy of the model's predictions, with a larger area under the curve (AUC) indicating a higher accuracy of the model. Calibration plots were usually used to assess the degree of fit between the predicted and actual scenarios; the higher the overlap between the predicted and actual curves, the closer the predicted scenario to the actual scenario.

We used internal validation to verify the stability of the model. A third of the patients were randomly selected by R as an internal validation cohort and the model was likewise assessed by ROC curves and calibration plots. All statistics were analyzed by SPSS 22.0 and R version 4.0.

## Results

A total of 367 eligible patients were included in the study cohort. Among the 367 patients, 38.3% were women and 61.7% were men, with a mean age 61.8 years. The differences between the PSCI and non-PSCI patients are highlighted in Table 1. At the 3 month follow-up, 87 out of the 367 patients were diagnosed with PSCI (23.7%). Compared with the non-PSCI patients, PSCI patients were more likely to be women (32.90 vs. 57.50%) and older (60.12 vs. 67.25). They also showed significant differences in stroke severity on admission, with a mean NIHSS score of 3.94 for the PSCI patients and 2.40 for the non-PSCI patients.

**Table 1:** Clinical and demographic characteristics of the samples under study.

	**Non-PSCI**	**PSCI**	***P* value**
Age	60.12 ± 10.25	67.25 ± 8.80	<0.001
NLR	2.14 ± 0.80	2.73 ± 1.26	<0.001
BMI	24.89 ± 7.93	23.48 ± 3.28	0.028
HbA1c	6.58 ± 1.79	6.32 ± 1.39	0.566
TC	4.69 ± 1.16	4.81 ± 1.15	0.424
TG	1.92 ± 1.35	1.58 ± 0.58	0.294
HDL	1.09 ± 0.27	1.18 ± 0.29	0.003
LDL	2.72 ± 0.95	2.81 ± 0093	0.332
LAA	72.90%	75.90%	0.677
NIHSS	2.40 ± 1.64	3.94 ± 2.50	<0.001
Male	67.10%	42.50%	<0.001
Hypertension	70.70%	75.9%%	0.413
DM	25.00%	20.70%	0.473
Coronary disease	7.60%	8.00%	1.000
Hyperlipidemia	11.50%	9.20%	0.695
Stroke history	9.00%	14.90%	0.157
Smoke	48.20%	43.70%	0.539
Drink	35.70%	32.20%	0.607
MMSE	22.20 ± 3.67	21.71 ± 3.08	0.264
Diseases of the circulatory system	71.80%	78.20%	0.240
Diseases of the nervous system	9.00%	14.90%	0.110
Endocrine, nutritional, and metabolic diseases	31.80%	25.30%	0.249
Mental and behavioral disorders	52.50%	57.50%	0.417

*NLR, neutrophil-to-lymphocyte ratio; BMI, body mass index; TC, total cholesterol; TG, triglyceride; HDL, high density lipoprotein; LDL, low density lipoprotein; DM, diabetes mellitus; LAA, large atherosclerotic infarction; NIHSS, National Institutes of Health Stroke Scale on admission; MMSE, Mini Mental State Examination. Table 1 shows the results of the univariate analysis of the patients' baseline data. Variables that are statistically different between the two groups (P ≤ 0.05) will be included in the logistic regression. There is no statistical difference in MMSE between the two groups of patients at baseline. Patients' comorbidities are classified according to ICD-10 criteria*.

**Figure 1:**
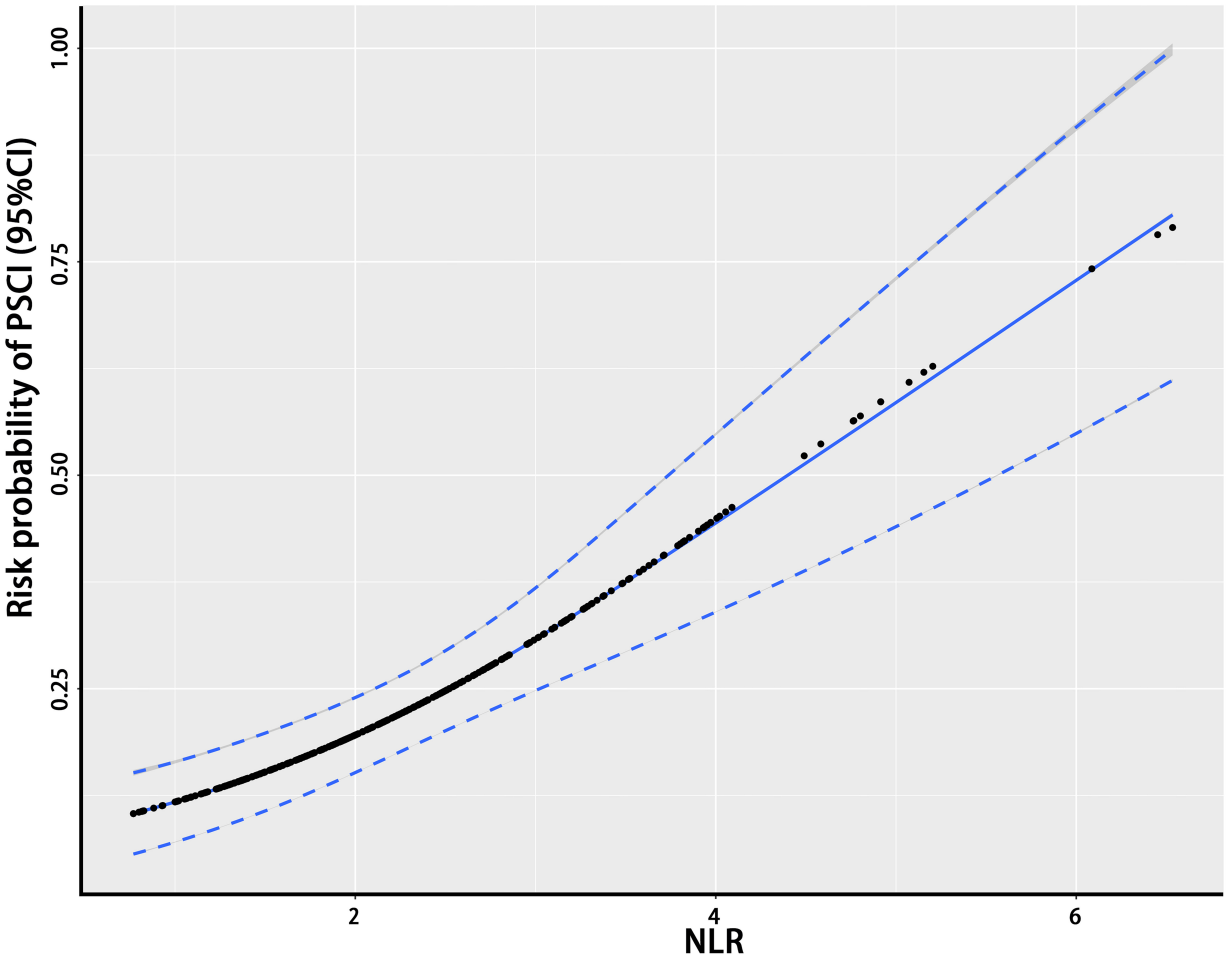
The relationship between the NLR and the probability of PSCI. This figure shows the association between the NLR and the probability of PSCI. The black dots represent the probability of the occurrence of PSCI corresponding to the given NLR level and the blue curves on either side are their 95% confidence intervals. NLR, neutrophil-to-lymphocyte ratio; PSCI, poststroke cognitive impairment.

**Figure 2:**
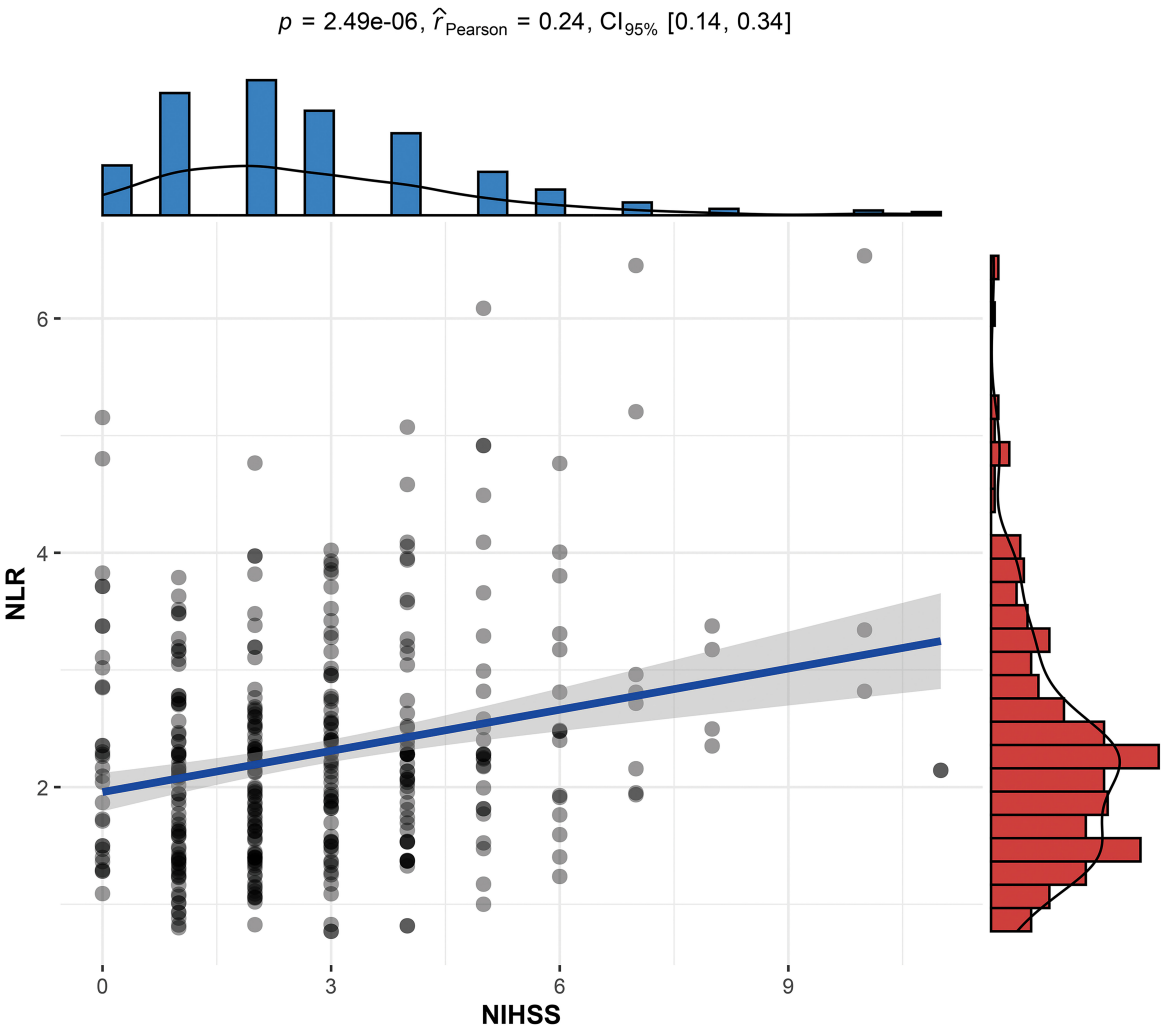
Linear correlation between the stroke patient's NLR and NIHSS score. NLR, neutrophil-to-lymphocyte ratio; NIHSS, National Institutes of Health Stroke Scale on admission; PSCI, poststroke cognitive impairment.

The NLR, the hypothetical marker in this paper, also showed significant differences between the PSCI and non-PSCI patients. As shown in Table 1, the mean NLR for the PSCI patients was 2.73, compared to 2.14 for the non-PSCI patients. The probability of PSCI was also closely linked to the NLR. Figure 1 shows the risk probability of PSCI (95 CI%). When a patient's NLR was elevated, then the probability of that patient developing PSCI was subsequently elevated. In addition to this, the NLR showed a strong correlation with stroke severity, as shown in Figure 2, where patients' NLR on admission was correlated with their NIHSS score (*P* < 0.001, *r* = 0.24, 95% CI: 0.14–0.34).

**Figure 3:**
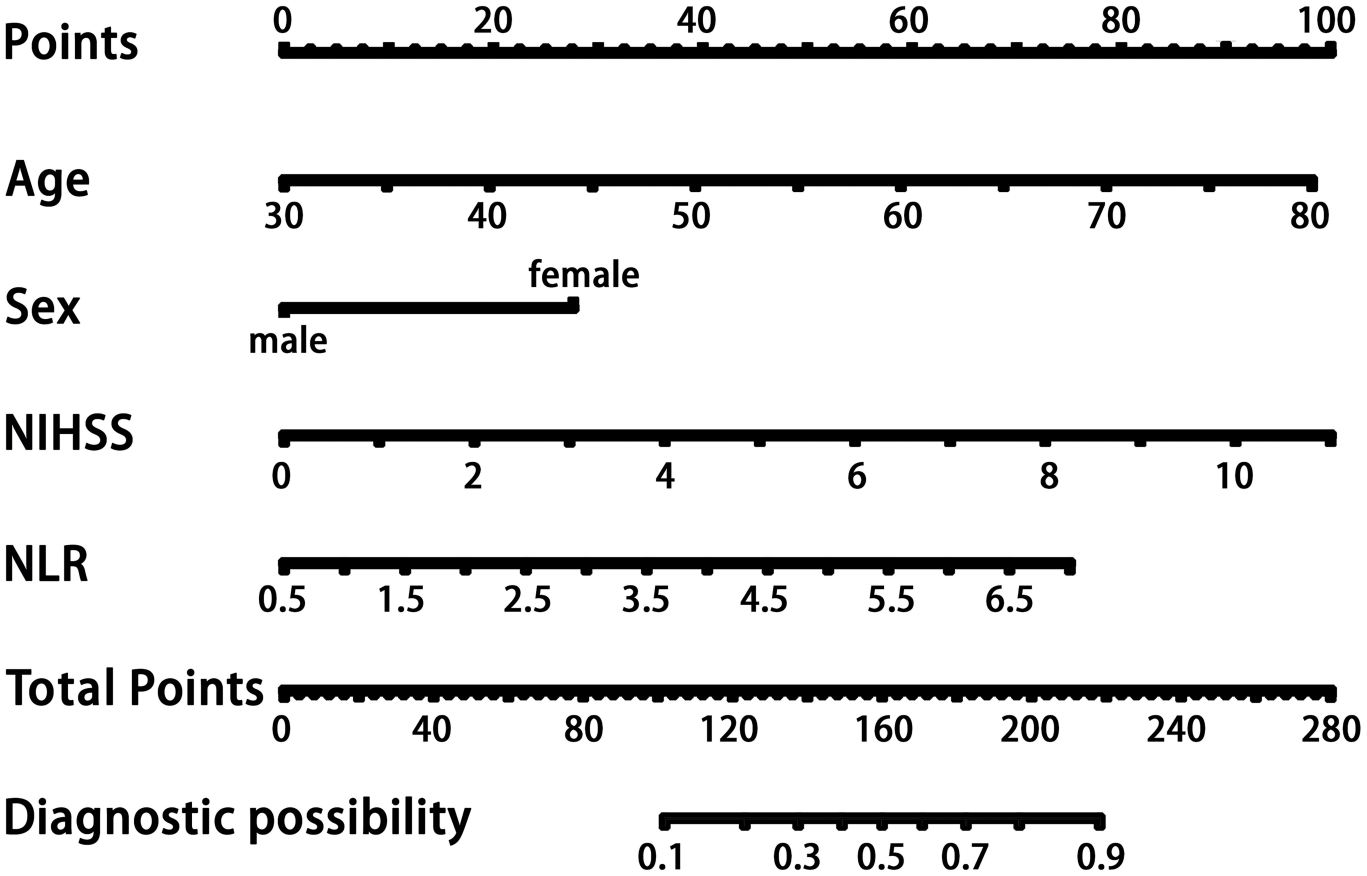
The nomogram for patients with PSCI. Each variable corresponds to its own score, and the sum of these scores corresponds to the probability of PSCI occurring on the probability axis. NLR, neutrophil-to-lymphocyte ratio; NIHSS, National Institutes of Health Stroke Scale on admission; PSCI, poststroke cognitive impairment.

In the logistic regression analysis, after adjusting for multiple confounding factors (age, sex, high-density lipoprotein level, NIHSS score, body mass index), higher NLR values were independently associated with the occurrence of PSCI at 3 months with an adjusted OR of 1.67 (95%CI: 1.21–2.29, *P* = 0.002). Additionally, patients' age (OR = 1.06, 95%CI: 1.03–1.10, *P* = 0.001), sex (male as the reference, OR = 2.94, 95%CI: 1.58–5.45, *P* = 0.001), and NIHSS score at admission (OR = 1.42, 95%CI: 1.22–1.64, *P* < 0.001) remained independently associated with the occurrence of PSCI (Table 2). MMSE at baseline may have an impact on cognitive function at the 3 month follow-up, so we independently explored its impact on the final outcome. As shown in Table 1, there was no difference in MMSE at baseline between the two groups of patients in the univariate analysis. As shown in Table 2, in model 2 we performed logistic regression analysis on the variables that were statistically different in the univariate analysis described above. To further control the MMSE, we included it in model 1. As shown in model 1, the MMSE did not show a statistical difference in the logistic regression analysis (P: 0.769, OR: 1.013, 95% CI: 0.930–1.103; Z-MMSE: P: 0.769,OR: 1.05,95%CI: 0.77–1.42). And, the OR of NLR changed from 1.63 to 1.60, showing a limited effect of baseline MMSE on NLR. After controlling for MMSE, NLR remained strongly associated with PSCI (P: 0.003, 95%CI: 1.18–2.25; Z-NLR: P: 0.003, 95%CI: 1.17–2.18). Hence, baseline MMSE was not included in the nomogram. Finally, the four predictors screened by the regression analysis were incorporated into the nomogram to predict the probability of PSCI occurrence. Figure 3 shows the nomogram, which was constructed by incorporating the four predictors. As shown, each predictor has its own corresponding score, and the sum of the scores of these factors corresponds to the probability of PSCI occurring in this case.

**Table 2:** Logistic regression analysis of risk factors for suffering from PSCI.

	**OR**	**95%CI**	***P* value**
Age	1.06	1.03–1.10	0.001
NLR	1.67	1.21–2.29	0.002
BMI	0.95	0.86–1.04	0.255
HDL	1.64	0.60–4.53	0.338
NIHSS	1.42	1.22–1.64	<0.001
Male	2.94	1.58–5.45	0.001
Z-age	1.89	1.31–2.73	0.001
Z-NLR	1.63	1.20–2.21	0.002
Z-BMI	0.68	0.34–1.33	0.255
Z-HDL	1.15	0.87–1.52	0.338
Z-NIHSS	2.00	1.50–2.67	<0.001
Male	2.94	1.58–5.45	0.001

*NLR, neutrophil-to-lymphocyte ratio; BMI, body mass index; HDL, high density lipoprotein; NIHSS, National Institutes of Health Stroke Scale on admission; PSCI, poststroke cognitive impairment. The logistic regression analysis of simple and standardized continuous variables (prefix with Z) is shown in the table. The OR of the former represents the change in the probability of occurrence of PSCI per unit increase, while the latter represents the change in the probability of occurrence of PSCI per standard deviation increase. Variables that were statistically different between the two groups (age, NLR, NIHSS, sex) were finally included in the nomogram*.

**Figure 4:**
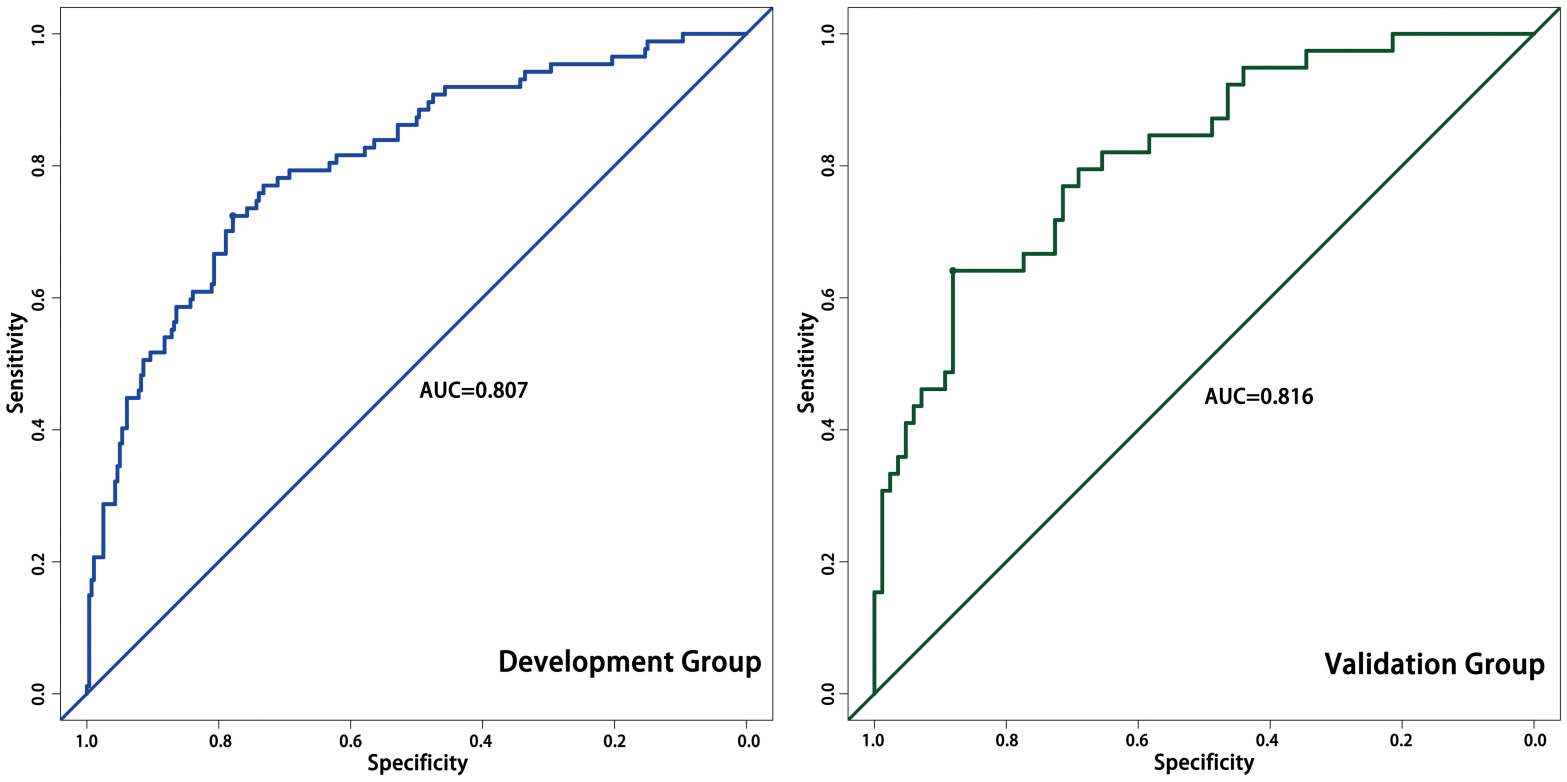
The ROC curves of the development cohort and validation cohort.

**Figure 5:**
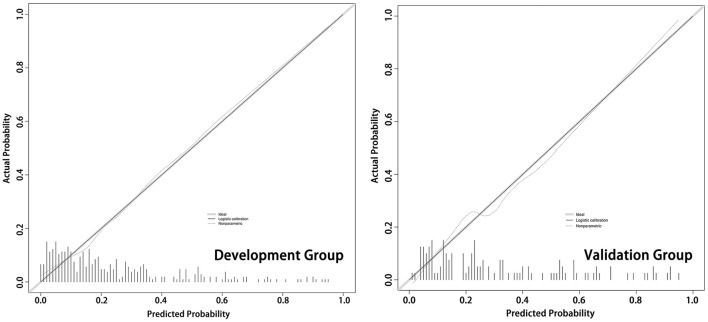
The calibration plots of the development cohort and validation cohort. As shown in the calibration plots, the predicted probability of the model is plotted on the x-axis; the actual overall event is plotted on the y-axis. The higher the degree of overlap between the predicted curve and the diagonal (actual situation), the more accurate the model.

We assessed the accuracy of the nomogram by ROC curves. In the development group, the AUC was 0.807, demonstrating good predictive power. Internal validation was used to assess the stability of the model. In the internal validation, the AUC of the model was 0.816, which was close to that of the development group, again demonstrating good predictive power (Figure 4). Except for the AUC, calibration plots are one of the tools that are commonly used to assess the consistency of a predicted situation against an actual situation. As shown in Figure 5, the predicted curves showed the best agreement with the actual curves in both the development and validation groups.

## Discussion

Patient sex, age, and the NIHSS score were considered to be associated with the occurrence of PSCI in previous studies (29–31). Few studies have considered using the NLR as an easily available peripheral inflammatory factor, and its relationship to PSCI (19). Our results suggest that higher NLR levels at admission are associated with the presence of PSCI. This is the first study to build a nomogram on the PSCI, filling a gap in this area.

In a review by Sun et al., demographically, age and literacy were associated with the risk of cognitive impairment after stroke (29). Age is a risk factor not only for stroke, but also for cognitive decline. There is evidence that the prevalence of cognitive decline after stroke increases exponentially with age after the age of 65 years (1). In a study by Chen et al., female sex was identified as a risk factor for cognitive decline (30). Sharma et al. found a strong correlation between the occurrence of PSCI and the patient's NIHSS (31).

A considerable amount of literature has already reported that cognitive impairment or dementia is related to inflammatory factors (14, 32–34). Diniz et al. found that increased expression of sTNFR1, which represents the TNF-α signaling system, is associated with a higher risk of progression from MCI to AD (35). Patanella AK et al. investigated the differential impact of BDNF and IL-6 on cognitive impairment and showed that low BDNF and high IL-6 levels are associated with poor performance in cognitive tasks in patients with relapsing-remitting multiple sclerosis. Although these inflammatory factors show significant correlations with cognitive function, these markers are uncommon in clinical work and often require additional testing to obtain them. Therefore, the NLR was selected as a target marker for this study as a readily available and clinically used peripheral inflammatory factor. In our study, we found that the NLR was positively correlated with stroke severity at admission and that high NLR levels predicted a higher likelihood of PSCI.

The NLR reflects the balance between neutrophil and lymphocyte levels and is a widely available, easily derived, and reproducible marker of inflammation. In accordance with the present results, previous studies have emphasized that the NLR is associated with atherosclerosis and is an independent risk factor for ischemic stroke (36, 37). Studies over the past decade have provided valuable information about the NLR and cognitive impairment or dementia. Hadi et al. found that the NLR at admission is associated with cognitive dysfunction after carotid endarterectomy (26). Mehmet et al. found that the NLR has a high sensitivity, specificity, and predictive value for AD patients (25). Due to the multifactorial pathophysiology of poststroke cognitive decline, the precise mechanisms remain unclear. Vascular mechanisms, which include lesions on small cerebral vessels, lead to cerebral microbleeds (CMBs), and white matter lesions may be the pathogenesis of PSCI (29, 38). Extensive research has shown that when the brain develops microbleeds, it has an inflammatory response to the hemorrhage (39–42). Inflammatory cytokines, including IL-6, IL-10, CRP, MCP-1, ICAM-1, and TNF-α, play an important role in the development of poststroke cognitive decline (14, 15, 43). One study reported that the NLR is positively correlated with inflammatory cytokines (44). Another study, a correlation analysis showed that hsCRP was positively correlated with the NLR in patients with Behcet disease (24). As a marker of inflammation, anti-inflammatory treatment can reduce neuroinflammation, AD-like pathology, and cognitive deficits (45).

Animal models with disturbed endothelial dysfunction have demonstrated that blood-brain barrier (BBB) breakdown either initiates or exacerbates neurodegeneration, which could lead to PSCI (46). Destruction of the BBB can disrupt the flow of oxygen and nutrients to the brain and allow toxins from around the brain to enter the central nervous system (47, 48). The NLR is a marker of endothelial dysfunction (49), and the accumulation of neutrophils may cause chronic BBB damage (50). Neutrophils have been shown to be an important source of matrix metalloprotein-9 that can breakdown the BBB or be absorbed by endothelial cells and act on the basement membrane (51). In a rat model of cerebral ischemia, treatment to prevent neutrophil infiltration reduces the release of matrix metalloprotein-9 in the brain (52). Moreover, the infiltration of neutrophils in the central nervous system can induce a cascade of neuroinflammatory reactions, aggravating cognitive decline. This shows that the NLR is closely related to the development of PSCI.

There are still several limitations in this study. First, this was a retrospective study and is subject to recall bias. During the follow-up process, selection bias was an inevitable result as a proportion of patients did not meet the inclusion criteria and were excluded. Second, this study used a single center's internal validation to verify the accuracy of the model, and whether the model can be generalized to other levels of medical centers is still not well validated. Despite its limitations, the highlight of this study is the creation of a relevant nomogram for PSCI patients by using a sample of a certain amount of data, which has not been done before. The indicators that were selected are also clinically accessible, so that clinicians can easily determine the likelihood of PSCI in a particular stroke patient and take appropriate precautions for it.

## Conclusion

This study suggests that a higher NLR may be associated with the occurrence of PSCI, and the constructed nomogram can help predict the probability of PSCI in stroke patients.

## Data Availability Statement

The raw data supporting the conclusions of this article will be made available by the authors, without undue reservation.

## Ethics Statement

The studies involving human participants were reviewed and approved by the Ethics Committee of the second people's Hospital of Hefei. Written informed consent for participation was not required for this study in accordance with the national legislation and the institutional requirements.

## Author Contributions

LY designed this study. FZ participated in the design and completed the statistical part of this article. CC, XJ, ML, and YW participated in the collection of data and the writing of the article. All authors contributed to the article and approved the submitted version.

## Conflict of Interest

The authors declare that the research was conducted in the absence of any commercial or financial relationships that could be construed as a potential conflict of interest.

## Publisher's Note

All claims expressed in this article are solely those of the authors and do not necessarily represent those of their affiliated organizations, or those of the publisher, the editors and the reviewers. Any product that may be evaluated in this article, or claim that may be made by its manufacturer, is not guaranteed or endorsed by the publisher.
